# Production, Biochemical Characterization, and Application of Laccase from Halophilic *Curvularia lunata* MLK46 Recovered from Mangrove Rhizosphere

**DOI:** 10.3390/biology14040402

**Published:** 2025-04-11

**Authors:** Malak Alshammary, Essam Kotb, Ibtisam M. Ababutain, Amira H. Alabdalall, Sumayh A. Aldakeel, Sumayah I. Alsanie, Salwa Alhamad, Hussah Alshwyeh, Ahmed M. Albarrag

**Affiliations:** 1 Department of Biology, College of Science, Imam Abdulrahman Bin Faisal University (IAU), P.O. Box 1982, Dammam 31441, Saudi Arabia; 2230500263@iau.edu.sa (M.A.); iababutain@iau.edu.sa (I.M.A.); aalabdalall@iau.edu.sa (A.H.A.); sualsanea@iau.edu.sa (S.I.A.); smalhamad@iau.edu.sa (S.A.); haalshuyeh@iau.edu.sa (H.A.); 2 Basic and Applied Scientific Research Center (BASRC), Imam Abdulrahman Bin Faisal University (IAU), P.O. Box 1982, Dammam 31441, Saudi Arabia; 3 The National Center for Genomic Technology (NCGT), Life Science and Environment Research Institute, King Abdulaziz City for Science and Technology (KACST), Riyadh 12354, Saudi Arabia; sadakeel@pha.gov.sa; 4 Genomic of Infectious Diseases Laboratory, Public Health Laboratory, Public Health Authority, Riyadh 13352, Saudi Arabia; 5 Department of Pathology, School of Medicine, King Saud University, Riyadh 12354, Saudi Arabia; aalbarrag@ksu.edu.sa

**Keywords:** laccase, fungi, pollutant, biodegradation, environmental sustainability

## Abstract

Laccase production was evaluated in 108 fungal isolates recovered from the eastern coast of Saudi Arabia, a critical element in environmental biodegradation and biotransformation. The most active isolate was identified as *Curvularia lunata* MLK46 (GenBank accession no. PQ100161). It exhibited maximal productivity at pH 6.5, 30 °C, and incubation for 5 d, with 1% sodium nitrate and 1% galactose as the preferred nitrogen and carbon sources, respectively. Productivity was enhanced by NaCl, CuSO_4_, and FeCl_3_ supplementation, with a maximum at 0.3 mM, 0.2 mM, and 61.7 mM concentrations, respectively. Sodium dodecyl sulfate polyacrylamide gel electrophoresis (SDS-PAGE) for the purified enzyme through diethylaminoethyl (DEAE)-Sepharose chromatography revealed a prominent band at 71.1 kDa with maximum activity at pH 6 and stability at pH 6–9. Furthermore, it was optimally active at 50 °C and thermally stable at 50–80 °C with a half-life time (*T*_1/2_) of 333.7 min to 80.6 min, respectively. Its activity was also enhanced by many metallic ions, especially Fe^3+^ ions; however, it was inhibited by Hg^2+^ and Ag^+^ ions. The enzyme demonstrated significant degradation of specific substrates such as 2,2’-azino-bis(3-ethylbenzothiazoline-6-sulfonic acid) (ABTS), guaiacol, *o*-dianisidine, and 2,6-dichlorophenol, with a kinetic efficiency constant which ranged from 40.95 mM^−1^ s^−1^ to 238.20 mM^−1^ s^−1^. UV spectrophotometry confirmed efficient oxidation peaks by electron transition against guaiacol (at 300 nm), *o*-dianisidine (at 480 nm), ABTS (at 420 nm), and 2,6-dichlorophenol (at 600 nm). The results collectively demonstrate the potential of laccase from *C. lunata* MLK46 as a promising agent for the effective biodegradation of several industrial pollutants under extreme conditions.

## Simple Summary

Industrialization including petrochemical and refinery sectors has adversely affected the quality of many coastal and terrestrial regions in many countries. The industrial effluents have prompted concerns about the health of living organisms and the proper functioning of ecosystem. Our hypothesis is that applying biological agents such as fungal laccases to degrade these contaminants may be a viable way to address this problem because of their adaptability, effectiveness, and low cost. Our results show that the isolated fungus *C. lunata* MLK46 from a marine environment exhibited exceptional productivity, and its oxidative enzyme demonstrated stability under extreme conditions. It also showed high catalytic efficiency in the degradation of several pollutants, positioning it as an eco-friendly and sustainable alternative to conventional chemical methods for bioremediation and environmental pollution control.

## 1. Introduction

Globally, the development of industry and agricultural practices adversely affects the environment, as effluents frequently contain mixtures of pollutants, including heavy metals, pesticides, dyes, and other hazardous substances. In parallel, the Arabian Gulf countries, specifically Saudi Arabia, have undergone tremendous industrialization and urbanization, especially in manufacturing industries, refineries, and petrochemicals. As a result, the coastal regions and waterways are heavily impacted. The discharge of untreated or poorly treated industrial effluents has prompted concerns about the health of marine ecosystems and seafood safety [1]. The increasing industrial activities in the region, particularly in the petrochemical and refinery sectors, contribute to high levels of pollutants in the environment, making the study of pollution mitigation methods even more crucial. Therefore, applying biological agents to detoxify or degrade these pollutants has become a viable and environmentally friendly way to address this problem. Fungal-produced enzymes, particularly laccases, have attracted a lot of interest among a wide range of biocatalysts used in bioremediation procedures because of their adaptability and effectiveness in breaking down these contaminants [2].

Laccases are multicopper oxidases (EC 1.10.3.2) with a glycoprotein nature due to their variable carbohydrate and copper content, and they are expressed by many genes. They catalyze the oxidation of a variety of substances, such as lignin, aromatic amines, and other xenobiotics, and are important biocatalysts for bioremediation applications because of their oxidation capabilities in both phenolic and non-phenolic substrates [3]. They are multifunctional enzymes used in a variety of fields, including environmental and industrial applications, notably in the bioremediation of industrial waste, and are useful for the detoxification of industrial wastewater and polluted soils due to their broad substrate specificity, which includes certain synthetic dyes, and polycyclic aromatic hydrocarbons. Their application in decolorization of dyes, as well as degradation of lignin, lies within the constraints of green chemistry principles. Laccases can be used in detoxifying pesticides and pharmaceutical waste residues that contribute to global environmental issues and are resolutely eco-friendly and energy-efficient [2,4].

In the presence of molecular oxygen, laccases can efficiently oxidize the phenolic substrates *o*- and *p*-phenols, methoxy phenols, phenolic acids, aromatic amines, and other aromatic structures, establishing phenoxy free radicals, which spontaneously rearrange to open the benzene rings or induce their polymerization [5]. Laccases are superior to peroxidases in that they only require the presence of their substrates and O_2_ as the electron acceptor. Furthermore, they have a wider range of substrates, more stability, non-inhibition in the presence of H_2_O_2_, and the non-liberation of any toxic peroxide intermediates. These characteristics make them most attractive and appropriate for applications in oxidative industrial processes [6].

Fungi are recognized as the most prolific producers of laccases and are extensively studied for biotechnological applications. Among them, the white-rot fungi *Pycnoporus sanguineus*, *Pleurotus ostreatus*, *Trametes trogii*, *Trametes versicolor*, *Fomes fomentarius*, *Ganoderma lucidum*, and *Pycnoporus cinnabarinus* are the most common producers. Additionally, laccases have been produced from higher plants, animals, insects, and some bacteria, including actinomycetes and *Bacillus* species. However, these have lower redox potential, and the produced amounts are lower compared to fungi. Productivity by microorganisms can be optimized through various fermentation techniques using various nitrogen and carbon sources [1,6]. Nevertheless, the substrate specificity of laccases distinctly varies based on the producing microorganism [7]. Catalytic efficiency measures the effectiveness of a laccase in the biotransformation of a specified substrate to a given product. According to published research, there is a considerable variance in the measured *K*_cat_, *K*_m_, and *K*_cat_/*K*_m_ among laccases [6].

Halophilic laccases are particularly valued for their stability and catalytic efficiency under extreme conditions, such as high salinity and elevated temperatures. These enzymes have proven efficient in bioremediation, particularly in degrading pollutants in hypersaline wastewater. They also support biofuel production by enhancing lignin degradation and improving biomass conversion. Additionally, their stability in organic solvents makes them suitable for pharmaceutical synthesis and biopolymer modification, emphasizing their broad industrial potential in pollution mitigation and sustainable manufacturing. The study of halophilic fungi is critical for understanding stress adaptation mechanisms and evaluating the biotechnological potential of their metabolites. These fungi, which thrive in saline environments such as mangrove forests and coastal regions, possess properties that make their enzymes highly relevant for industrial applications. The Qatif Coast of Saudi Arabia, with its elevated salinity levels and proximity to industrial pollution, provides a distinct ecological niche for these fungi. However, there is a notable lack of research focused specifically on halophilic fungi in the Arabian Gulf region, particularly concerning their potential for pollution mitigation and bioremediation in saline environments. Given their enzymatic activities, these fungi may play a significant role in pollutant degradation, thus positioning the Qatif Coast as a valuable, yet underexplored, source of halophilic fungi with potential bioremediation applications [8].

This study investigates the production, biochemical characterization, and potential applications of laccase derived from local halophilic fungi such as *C. lunata* strain MLK46, isolated for the first time from a polluted mangrove forest soil at Qatif Coast in the Arabian Gulf. The producing fungus exhibited exceptional productivity, and its enzyme demonstrated stability under extreme conditions and high catalytic efficiency in the degradation of several substrates, positioning it as an eco-friendly and sustainable alternative to conventional chemical methods for bioremediation and environmental pollution control.

## 2. Materials and Methods

### 2.1. Materials and Reagents

ABTS, bromophenol blue (BPB) stain, lactophenol cotton blue stain, EZBlue stain, potato dextrose agar (PDA), DEAE-Sepharose CL-6B, ethylenediamine-tetraacetate (EDTA), SDS, L-cysteine, 1,4-dithiothreitol (1,4-DTT), sodium azide (NaN_3_), 2-mercaptoethanol, 2,6-dichlorophenol, guaiacol, α-naphthol, and *o*-dianisidine were purchased from Sigma-Aldrich (St. Louis, MO, USA). Other analytical grade reagents were obtained from local suppliers.

### 2.2. Sampling and Isolation of Fungi

A survey for fungal isolates producing laccases in the eastern region of Saudi Arabia was conducted from March to August 2023, covering Dammam, Dhahran, Jubail, Tarout Island, and Khobar. Soil samples (50–100 g) were collected from various habitats using a sterilized shovel, targeting surface and subsurface layers to cover fungal diversity across soil profiles. Liquid samples were obtained from water bodies using sterile containers, with collections made at the surface and at different depths to represent fungal diversity throughout the water column. The serial dilution method was applied to decrease the cell concentration to the acceptable limit. Once the dilutions were prepared, 1 mL from each aliquot was transferred into petri dishes with approximately 20 mL of PDA, which were then incubated at 30 °C for 5 d to allow the growth of fungi.

### 2.3. Screening for Potent Laccase Producers

The ABTS plate protocol initially conducted the qualitative screening for laccase productivity. For this, the ABTS agar medium was prepared from (g/L) agar 15, potato broth 8, dextrose 10, peptone 1, NH_4_Cl 4, K_2_HPO_4_ 1, FeSO_4_ 0.01, NaCl 0.1, CaCl_2_ 0.1, MgSO_4_·7H_2_O 0.5, CuSO_4_ 0.03, and ABTS 0.2. The formation of dark green areas around colonies was confirmatory for laccase production. The positive isolates were then checked for their ability to degrade the BPB stain. For this, the same medium components were prepared by replacing ABTS with the same concentration of 2,6-dichlorophenol, BPB [9]. Then, it was subjected to sterilization for 20 min at 121 °C. Using a sterilized scalpel, squares of about 20 mm^2^ were cut from plates containing fungal growth and then loaded onto the middle of assay plates. The pH was adjusted to 5.50, and incubation was performed at 30 °C for 5 d. Individual fungal colonies showing cleared halos around their growth were taken as positive producers. The selected isolates were cultured on PDA at 4 °C for short-term preservation and into 20% (*v*/*v*) glycerated nutrient broth at −80 °C for long-term preservation.

### 2.4. Enzyme Production and Assay Procedure

Inocula from the most potent isolates degrading both ABTS and BPB were then allowed to grow in basal production medium consisting of 0.10 g of yeast extract, 10.00 g of glucose, 0.22 g of (NH_4_)_2_SO_4_, 0.20 g of KH_2_PO_4_, 0.05 g of MgSO_4_, 0.50 g of KCl, 0.05 g of sodium molybdate, 0.03 g of FeSO_4_, 0.50 g of ZnSO_4_, and 0.08 g of CuSO_4_ per liter. Exactly 2% (*v*/*v*) of fungal spores (108 spore/mL) were applied to inoculate 20 mL of the liquid medium in 100 mL-volume Erlenmeyer flasks. The initial pH was fixed to 5.50, and incubation was performed at 30 °C for 7 d. After incubation, the liquid medium was filtered through Whatman Filter Paper No. 1 and then centrifuged (Universal 320 R, Hettich, Tuttlingen, Germany) at 5000 rpm for 20 min in a cooling centrifuge for the separation of crude enzyme. The quantitative laccase assay was performed by homogenizing 900 µL of the prepared enzyme with 100 µL of 30 mM ABTS. The reactants were incubated at 50 °C. Absorbance at 420 nm (*A*_420_) was measured after 1 min using a UV-1900i spectrophotometer (Shimadzu, Kyoto, Japan). A blank was set under the same conditions by replacing the enzyme with distilled water. Each unit of laccase activity was identified as the quantity of enzyme needed to release 1 mM of ABTS per mL per min at the standard reaction conditions.

### 2.5. Identification of the Potential Isolates

Fungi were stained with lactophenol cotton blue, and then their morphology was examined using a BX53 light microscope (Olympus, Tokyo, Japan). Isolates were molecularly identified after the extraction of their DNA. The QIAamp DNA Mini Kit (QIAGEN, Hilden, Germany) was used to extract the genomic DNA from fungal isolates, which was further homogenized with 0.5 mm glass beads and incubated overnight at 56 °C. A NanoDrop 2000c spectrophotometer (Thermo Fisher Scientific, Waltham, MA, USA) was applied to quantify the extracted DNA. The PCR amplification of the fungal ribosomal RNA gene cluster (*18S*, *5.8S*, and *28S* rRNA) ITS1 and ITS2 regions was performed using 2X GoTaq Green Master Mix (Promega, Madison, WI, USA) and primers ITS1 (5′-CTT GGT CAT TTA GAG GAA GTA A-3′) and ITS4 (5′-TCC TCC GCT TAT TGA TAT GC-3′). The amplicons were treated with ExoSAP-IT (Applied Biosystems, Foster City, CA, USA) for purification and subsequently sequenced on a SeqStudio Genetic Analyzer (Applied Biosystems, USA) by a Big Dye Terminator v3.1 Cycle Sequencing Kit (Thermo Fisher Scientific, Waltham, MA, USA).

### 2.6. Phylogenetic Analysis

Multiple-sequence alignment for selected fungi was performed using ClustalW with default parameters to align the sequences. The resulting alignment file was used to construct a phylogenetic tree. A neighbor-joining (NJ) tree was generated based on the aligned sequences. The generated tree file in Newick format was then uploaded to Interactive Tree of Life (iTOL; https://itol.embl.de/, accessed on 29 March 2025) for visualization and annotation. Branch support values were included, and the tree was customized using iTOL’s built-in features.

### 2.7. Upstream Processing of Laccase Production

The effect of key factors such as the fermentation temperature, period, pH, key minerals (NaCl, MnSO_4_, and CuSO_4_), nitrogen source, and carbon source were studied to accomplish the maximum laccase production from the most potent isolate. Each factor was examined independently to identify the conditions yielding the highest productivity. Once optimal results were obtained for a parameter, the study progressed to the next experiment.

### 2.8. Laccase Purification

Following filtration, the liquid culture was centrifuged at 5000 rpm for 20 min, then soluble proteins were salted out by gradual addition of ammonium sulphate with vigorous stirring until an 80% (*w*/*v*) concentration limit. The enzyme was dialyzed against distilled water at 4 °C to remove the nonprotein structures. The dialyzed proteins were eluted through a DEAE-Sepharose CL-6B column (1 × 15 cm) previously equilibrated with 50 mM sodium phosphate buffer (pH 6.0, buffer A) [10]. The Next Generation Chromatography (NGC) system (Bio-Rad, Hercules, CA, USA) was used in the separation process. The unbounded proteins were eluted with the same buffer with a linear rise in ionic strength of 20–800 mM (buffer B) at a 1 mL/min elution rate. Fractions were collected based on absorbance at 280 nm and analyzed for enzyme activity and purity. The molecular weight and homogeneity of MLK46 were assessed by SDS-PAGE analysis using a 4% stacking gel and a 12% resolving gel at a constant voltage of 200 V. Protein bands were stained by immersion in a ready-to-use Brilliant Blue G-250 (EZBlue) dye (Sigma-Aldrich, USA) for 5 min and destained by immersion in distilled water for another 5 min.

### 2.9. Biochemical Characterization of Laccase

#### 2.9.1. Effect of pH on Activity and Stability

Laccase fractions were adjusted to pH levels from 5.0 to 12.0. For activity measurements, 100 µL of 30 mM ABTS at the same pH value was mixed with 900 µL of the enzyme, and the reacting mixture was incubated at 50 °C for 1 min before measuring *A*_420_. For pH stability assessment, enzyme preparations were preincubated at 30 °C for 2 h under their specific pH values. Exactly 1 mL of 30 mM ABTS was added, and the remaining activity was then measured under the standard reaction settings.

#### 2.9.2. Effect of Temperature on Activity and Stability

For activity measurements, 100 µL of 30 mM ABTS was homogenized with 900 µL of enzyme preparations, and the reactants were incubated for 10 min at a temperature range of 40–100 °C to then measure *A*_420_. Thermal stability was assessed by preincubating the tested enzyme at 50–100 °C for 1 h. At 15 min intervals, fractions were withdrawn, and at the end of incubation, the remaining laccase activity was determined. Activity at 0 min was expressed as absolute activity (100%).

#### 2.9.3. Effect of Reagents

The influence of various reagents, including EDTA, SDS, L-cysteine, 1,4-DTT, NaN_3_, 2-mercaptoethanol, and hydrogen peroxide, on laccase activity was evaluated. The enzyme was preincubated at 30 °C for 2 h with each inhibitor at a final concentration of 0.2 mM, with 0 mM as the control. The remaining activity against ABTS was then measured.

#### 2.9.4. Effect of Metal Ions

The influence of metal ions such as Ca^2+^, Co^2+^, Fe^3^, Mn^2+^, Zn^2+^, and Cu^2+^ on laccase activity was evaluated. These were mixed with laccase preparations at final concentrations of 2, 4, 6, 8, and 16 mM, with 0 mM concentration serving as the control. These preparations were preincubated at 30 °C for 1 h, after which the remaining activity was assessed against ABTS as mentioned above.

#### 2.9.5. Substrate Specificity

The degradation of various compounds by MLK46 laccase was analyzed using a UV-visible spectrophotometer. A quartz cuvette with 1.0 cm length of path was used at a scanning rate of 0.1 nm and 1000 nm min^−1^. Tested substrates included 2,6-dichlorophenol, guaiacol, α-naphthol, ABTS, and *o*-dianisidine. The oxidation by electron transition was taken as an indication of breakdown, and the maximum absorbance in the oxidation peak was determined. Spectral scans were performed between 300 and 900 nm. For each substrate, 0.2 of a 30 mM solution at a fixed pH of 5.5 was mixed with 1.8 mL of laccase. The reactants were incubated at 30 °C for 2 h. A blank containing the substrate without the enzyme was included to ensure accuracy. The oxidation process was visualized for 10 min to detect the spectral shifts in the presence and absence of enzyme [6].

#### 2.9.6. Determination of Kinetic Constants

The dynamics of laccase were tested against different concentrations (0 mM to 400 mM) of the above substrates. Laccase activity was determined under the stated conditions, then the maximum velocity of reaction (*V*_max_) and the Michaelis–Menten constant (*K*_m_) were evaluated from the linear regression equation derived from the Lineweaver–Burk plot. The turnover number (*K*_cat_) was calculated from the ratio *V*_max_/*E*_t_, where *E*_t_ is the applied laccase concentration. Also, the specificity constant for each substrate was deduced from the ratio *K*_cat_/*K*_m_ and was then employed to determine the most preferred substrates for the tested laccase.

### 2.10. Statistical Analysis

Unless otherwise mentioned, the data throughout this study were statistically analyzed using Microsoft Excel 365 to evaluate enzyme activity. Data were presented as averages of three biological replicates ± standard deviations.

## 3. Results

### 3.1. Isolation and Screening of Laccase-Producing Fungi

**Figure 1:**
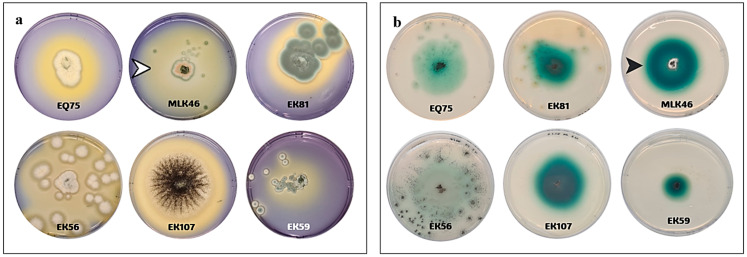
Comparable laccase productivity detected for some fungal isolates using bromophenol blue plate assay (**a**) and ABTS plate assay (**b**). The white arrowhead shows a yellow halo area due to the degradation of bromophenol dye, while the black arrowhead shows a green halo zone due to the degradation of ABTS by laccases produced from these fungi.

In this study, we have recovered 108 fungal isolates from different places in the eastern region of Saudi Arabia. The isolates were chosen depending on the morphological differences and reaction with the differential growth medium containing laccase substrate. Potent laccase-producing fungi were chosen based on their ability to degrade both ABTS and BPB stain. Yellow halos around the growing colonies were indicative of BPB degradation due to the secreted enzymes (Figure 1a). The green halos around the growing colonies on ABTS plates were indicative of laccase production (Figure 1b). From these qualitative assays and the quantitative ABTS assay, isolates EK12, MLK46, EK56, EK59, EQ75, EK81, and EK107 were selected as the most potent laccase producers (Table 1).

### 3.2. Characterization of Fungal Isolates

**Figure 2:**
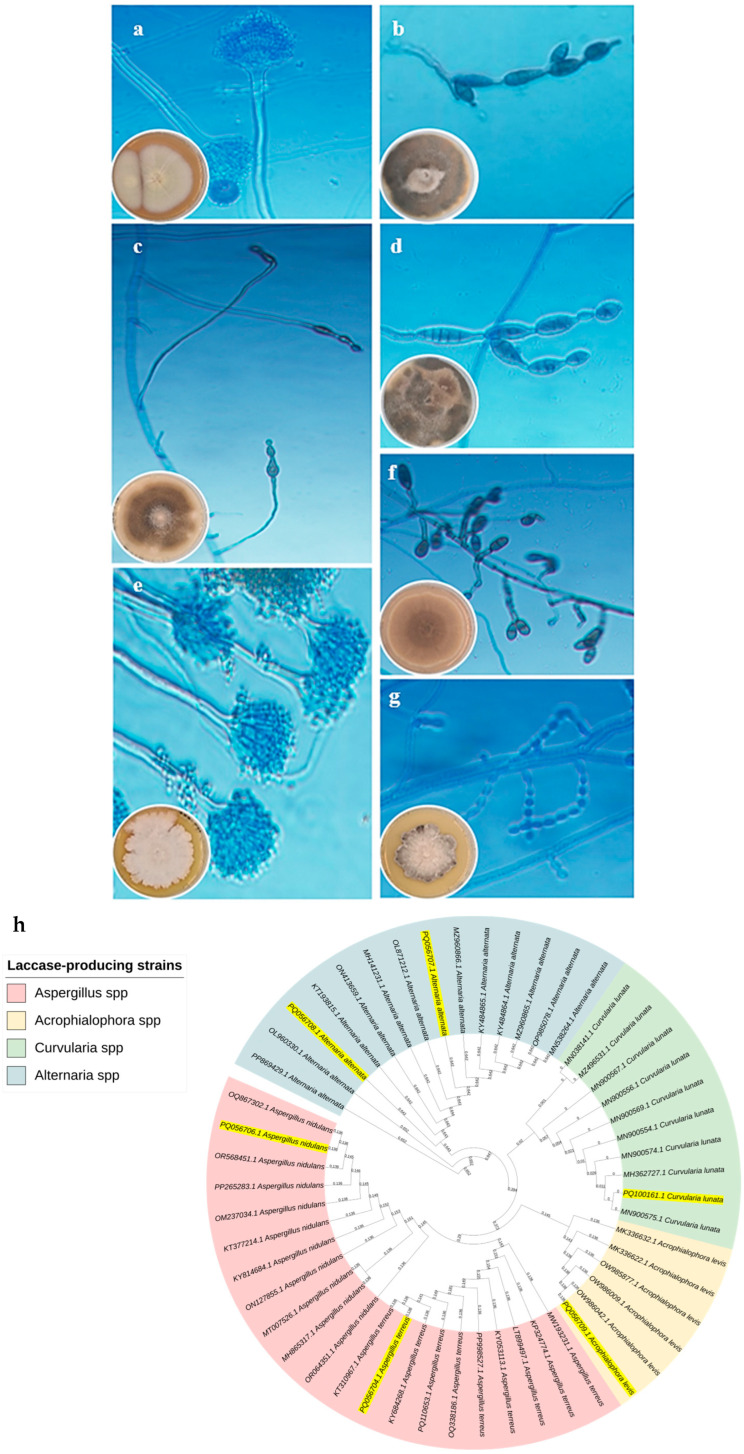
Macroscopic and microscopic structures of the most potent laccase producers stained with lactophenol cotton blue, highlighting distinctive features such as conidia, sporangia, and hyphal arrangements. The isolates include *A. terreus* EQ75 (**a**), *A. alternata* EK12 (**b**), *A. alternata* EK81 (**c**), *A. alternata* EK59 (**d**), *A. nidulans* EK107 (**e**), *C. lunata* MLK46 (**f**), and *Acrophialophora levis* EK56 (**g**). The phylogenetic tree of the most promising laccase-producing strain *C. lunata* MLK46 (accession number PQ100161.1) is shown in panel (**h**). The neighbor-joining phylogenetic tree shows the most promising fungal species isolated in this study, which are highlighted in yellow.

The morphological characteristics of the most promising fungal isolates were studied using macroscopic and microscopic observations at a magnification of ×40 (Figure 2a–g). They were then molecularly identified based on the 18S subunit ribosomal RNA gene, partial sequence; internal transcribed spacer (ITS) 1, 5.8S ribosomal RNA gene, and ITS 2, complete sequence; and 28S subunit ribosomal RNA gene, partial sequence fingerprint. The final characterization of the seven isolates was identified as *A. alternata* EK12 (accession number PQ106852.1), *C. lunata* MLK46 (accession number PQ100161.1), *Acrophialophora levis* EK56 (accession number PQ056709.1), *A. alternata* EK59 (accession number PQ056708.1), *A. terreus* EQ75 (accession number PQ056704.1), *A. alternata* EK81 (accession number PQ056707.1), and *A. nidulans* EK107 (accession number PQ056706.1) (Table 1). Figure 2h shows the phylogeny of these isolates with the closest strains in the GenBank database.

### 3.3. Optimization of Laccase Productivity

This involved systematic adjusting of various cultural and nutritional parameters, such as temperature, pH, nitrogen source, and carbon source. Table 2 describes the optimized parameters and the productivity in terms of enzymatic units (U/mL) and replication folds (*x*). The final laccase productivity by *C. lunata* MLK46 reached 324.3 U/mL, with a 3.4-fold increase compared to the preliminary unoptimized conditions (95.7 U/mL). It has been determined that the optimal pH was 6.5 (130.9 U/mL) and the optimal temperature was 30 °C (141.7 U/mL). In addition, productivity reached a peak on day five (211.7 U/mL) and subsequently exhibited a gradual decline. The best mineral supplementation was achieved with 1% (61.65 mM) FeCl_3_ (267.4 U/mL), 0.2 mM CuSO_4_ (257.83 U/mL), and 1.5% (0.26 mM) NaCl (221.36 U/mL) (Table 2). However, laccase production in the presence of the common medium ingredient MnSO_4_ showed minimal amounts with best productivity at 0.45% (0.3 mM, 116.1 U/mL). Maximum productivity was achieved with 1% (*w*/*v*) sodium nitrate (286.5 U/mL) followed by yeast extract, while malt extract was the least effective nitrogen source. Galactose at 1% (*w*/*v*) concentration was the best carbon source (324.3 U/mL) followed by fructose, while glucose and maltose showed significantly lower productivity.

### 3.4. Laccase Purification and Biochemical Properties

**Figure 3:**
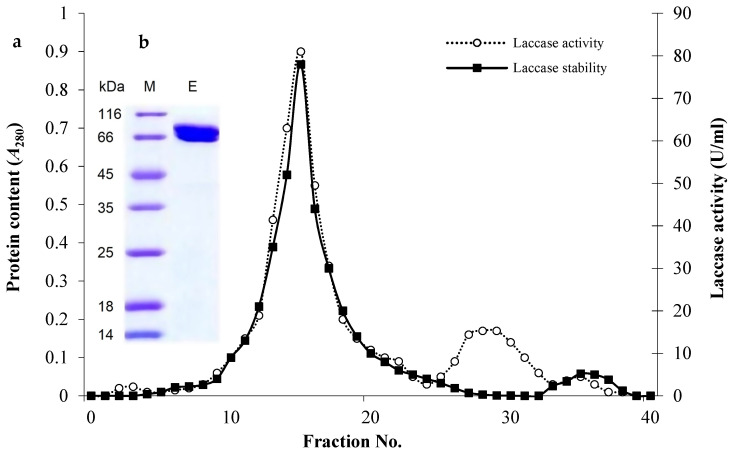
Chromatogram of the purified laccase from *C. lunata* MLK46 after elution through a DEAE-Sepharose column (1 × 15 cm) previously equilibrated with 50 mM sodium phosphate buffer (pH 6.0) (**a**). SDS-PAGE analysis of purified enzyme shows a distinct band at 71 kDa (**b**), indicating a successful purification procedure. Lane M represents the molecular weight markers, while lane E represents the purified protein.

**Figure 4:**
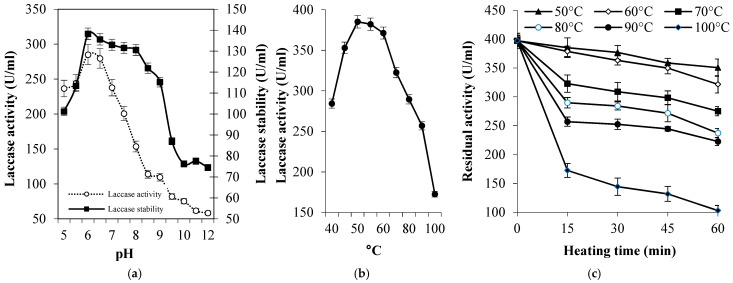
Effect of pH (**a**) and high temperatures on the activity (**b**) and stability (**c**) of tested laccase.

The eluted laccase through DEAE-Sepharose (Figure 3a) showed a major band with a molecular weight of 71.11 kDa on SDS-PAGE gel (Figure 3b). The single band confirms the homogeneity of the enzyme and suggests the monomeric nature of the purified laccase. The purified enzyme exhibited the maximum activity against ABTS at pH 6.0 and it was maximally pH-stable between pH 6.0–9.0 during the exposure time (2 h, Figure 4a). Furthermore, it was mostly active at 50 °C (Figure 4b) and exhibited remarkable thermal stability at elevated temperatures where the *T*_1/2_ required to reduce to half its initial values, where at 50 °C, 60 °C, 70 °C, 80 °C, 90 °C, and 100 °C these were 333.7 min, 197.2 min, 113.3 min, 80.6 min, 71.9 min, and 30.8 min, respectively (Figure 4c).

### 3.5. Inhibition Study

**Figure 5:**
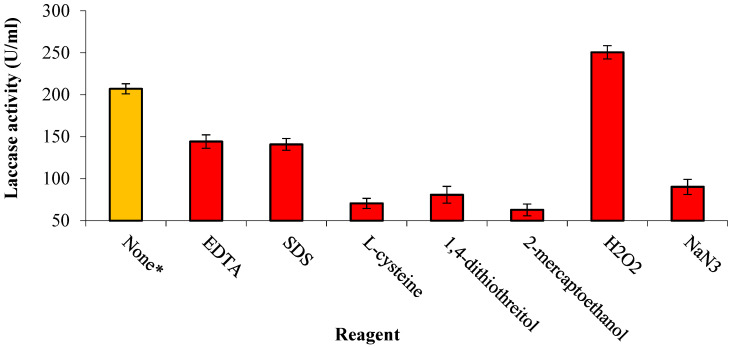
Effect of laccase reagents on enzymatic activity. The * symbol denotes the control preparation where no reagent was added to enzyme.

The impact of EDTA, SDS, L-cysteine, 1,4-DTT, NaN_3_, 2-mercaptoethanol, and H_2_O_2_ on the stability of laccase was measured and compared to that of the blank. Most reagents were inhibitors, while hydrogen peroxide was an activator (1.2-fold). The reagents 2-mercaptoethanol and L-cysteine exhibited the highest degree of inhibition, reducing the original enzyme activity to 69.6% and 65.9%, respectively (Figure 5).

### 3.6. Effect of Metal Ions on Laccase Activity

**Figure 6:**
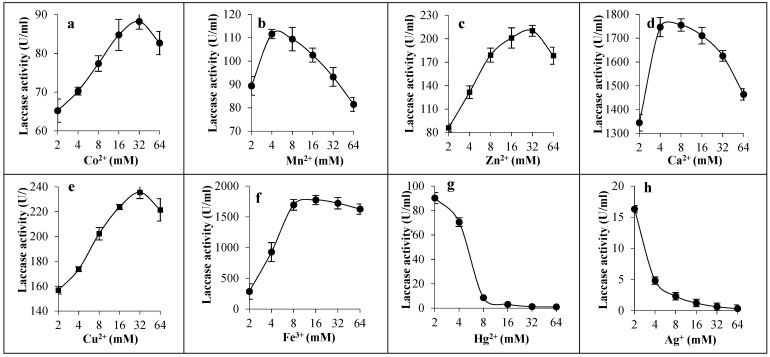
Effect of metal ions on the activity of laccase from *C. lunata* strain MLK46. The effect of cobalt (**a**), manganese (**b**), zinc (**c**), calcium (**d**), copper (**e**), ferric iron (**f**), mercury (**g**), and silver (**h**) were tested at the concentration range of 2–64 mM.

In the tested range of concentrations (2–64 mM), all metallic ions showed a stimulatory effect for laccase except Hg^2+^ and Ag^+^ ions. Most of the activity was lost at 8 mM of Hg^2+^ ions and at 2 mM of Ag^+^ ions, where the remaining activity was 8.6 U/mL and 16.3 U/mL, respectively. It was found that 32 mM concentration of Co^2+^ (88.25 U/mL), Zn^2+^ (210.3 U/mL), and Cu^2+^ ions (235.6 U/mL) was optimal for maximum laccase activity, while the concentration of 4–8 mM of both Mn^2+^ (110.59 U/mL) and Ca^2+^ ions was best (1755.07 U/mL). Furthermore, at 16 mM of Fe^3+^ ions the best activity of laccase was obtained (1773.9 U/mL). It can be concluded that Fe^3+^ and Ca^2+^ ions greatly contributed to the stimulation of activity when compared with other minerals (Figure 6).

### 3.7. Substrate Specificity and Kinetics Against Various Compounds

**Figure 7:**
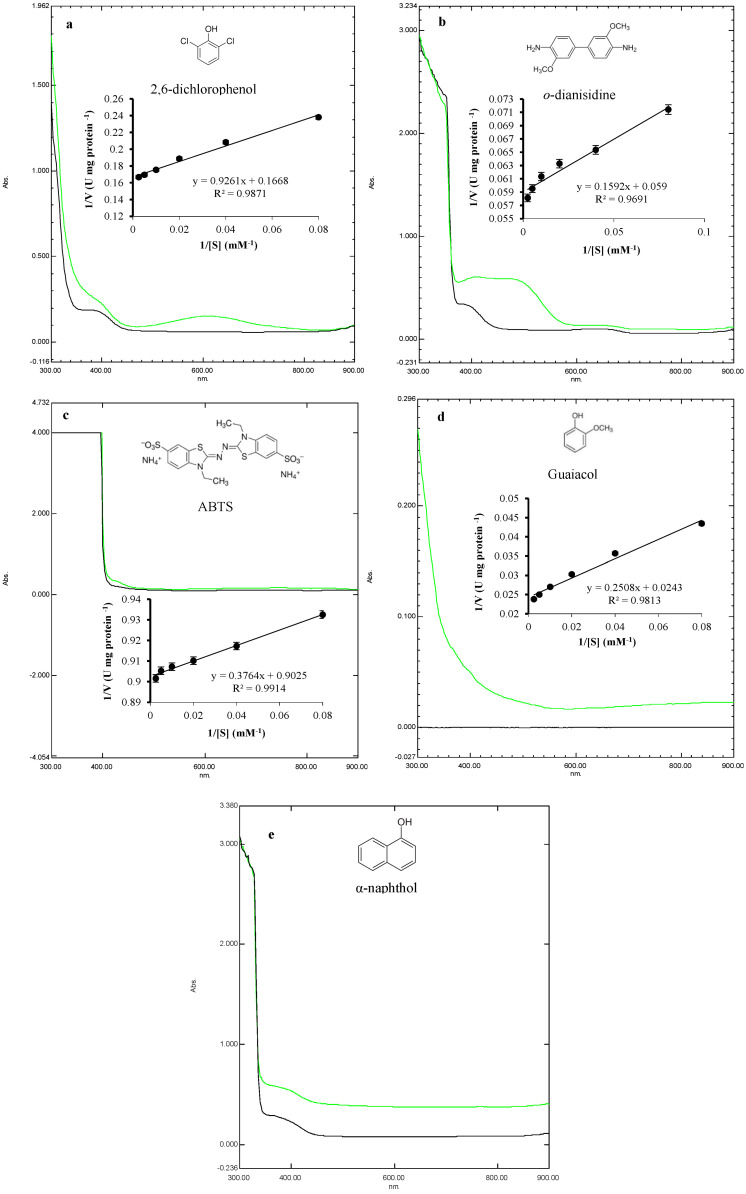
Spectral changes in the oxidation of various substrates by laccase. These substrates include 2,6-dichlorophenol (**a**), *o*-dianisidine (**b**), ABTS (**c**), guaiacol (**d**), and α-naphthol (**e**). The black lines represent the tested substrate without enzymatic treatment, while the green lines indicate substrates after laccase treatment. In the middle of the panels, the kinetic analysis of laccase activity against each substrate is represented in the form of Lineweaver–Burk plots.

A UV-visible spectrophotometer was used to compare the degradation efficiency of laccase by oxidation reaction. The utilized compounds included the phenolic substrates 2,6-dichlorophenol, guaiacol, and α-naphthol, in addition to the nonphenolic substrates *o*-dianisidine and ABTS. The spectral scans were performed between 300–900 nm (Figure 7). Spectral analysis revealed substrate-specific oxidation efficiencies of laccase through absorbance changes. In addition, 2,6-dichlorophenol showed characteristic oxidation peaks around 600 nm (Figure 7a), *o*-dianisidine showed a characteristic oxidation peak around 480 nm (Figure 7b), ABTS exhibited an oxidation peak at 420 nm (Figure 7c), and guaiacol showed the strongest oxidation peak around 300 nm (Figure 7d); meanwhile, the reaction of laccase with α-naphthol did not show any characteristic peaks (Figure 7e).

Furthermore, the catalysis of laccase against these specific substrates was plotted in the form of Lineweaver–Burk plots (Figure 7, middle of panels). When ABTS was the substrate, laccase exhibited a *K*_m_ of 4.17 mM and a *V*_max_ of 11.08 mM s^−1^. Furthermore, the *K*_cat_ was determined to be 420.19 s^−1^, with a catalytic efficiency of 100.75 mM^−1^ s^−1^. For guaiacol, laccase exhibited a *K*_m_ of 10.32 mM, a *V*_max_ of 41.15 mM s^−1^, a *K*_cat_ of 1560.57 s^−1^, and a catalytic efficiency of 151.20 mM^−1^ s^−1^. Against *o*-dianisidine, laccase exhibited the best dynamics where the *K*_m_ value was 2.70 mM, the *V*_max_ value was 16.95 mM s^−1^, the *K*_cat_ was 642.74 s^−1^, and the catalytic efficiency was 238.20 mM^−1^ s^−1^. Additionally, when 2,6-dichlorophenol was the substrate, laccase exhibited a *K*_m_ value of 5.55 mM and a *V*_max_ value of 6.00 mM s^−1^. Furthermore, the *K*_cat_ was determined to be 227.35 s^−1^, with a catalytic efficiency of 40.95 mM^−1^ s^−1^. Overall, most of the substrates were effectively oxidized by laccase, with the highest efficiency in the case of *o*-dianisidine, guaiacol, ABTS, and 2,6-dichlorophenol, respectively, as evidenced by the oxidation peaks and kinetic efficiency (*K*_cat_/*K*_m_) constant.

## 4. Discussion

Microorganisms, especially fungi, are known as potential degraders and a source of various hydrolytic and oxidative extracellular enzymes [10,11]. Unfortunately, most studied fungi were isolated from traditional terrestrial habitats, and few reports have investigated marine fungi for their enzymatic activity. The marine-derived strains may have evolved from their terrestrial counterparts, with the emergence of adaptability to high osmotic pressure, oligotrophic nutrients, etc. These conditions may explain the major variations between the enzymes produced from terrestrial-derived microorganisms and their marine counterparts [1,10,12].

Marine-derived laccases have distinct functional advantages, particularly in the high-salinity conditions of mangrove ecosystems. These, characterized by fluctuating salinity, tidal influences, and a combination of freshwater and seawater, create an extreme environment that most terrestrial organisms cannot tolerate. Marine fungi, such as *C. lunata* MLK46, have evolved enzymes capable of surviving these challenging conditions. Their laccases are inherently adapted to salinity fluctuations, making them uniquely suited to mangrove ecosystems where salinity can vary dramatically. This adaptation is critical for their ecological function and industrial applications in such environments. Moreover, marine-derived laccases display superior thermal stability, preserving enzymatic functionality over prolonged durations, a characteristic of significant relevance for industrial applications [13]. Laccases from halotolerant and halophilic fungi have emerged as promising biocatalysts due to their stability under high salinity, extreme pH, and elevated temperatures. These enzymes play a crucial role in bioremediation, wastewater treatment, dye decolorization, textile processing, and biofuel production by facilitating the degradation of lignin, synthetic dyes, and industrial pollutants [1].

Laccases exhibit broad substrate specificity, facilitating the oxidation of various organic compounds, including synthetic dyes and phenolic pollutants, thereby highlighting their potential in industrial and environmental applications. The distinctive physicochemical conditions of marine ecosystems drive structural and functional adaptations in these enzymes, enhancing their stability and catalytic efficiency under high-salinity and extreme environmental conditions. These properties are particularly relevant for pollution mitigation in hypersaline regions such as the Arabian Gulf. Moreover, laccases represent a promising alternative for wastewater treatment due to their ability to degrade a wide spectrum of contaminants [8].

Moreover, laccase production by fungal isolates recovered from unexplored habitats offers a great potential for the biodegradation of most industrial contaminants, as laccases are broad-spectrum biocatalysts capable of oxidizing different kinds of substrates and provide a cheap and renewable means for pollutant disposal. The coastal regions of the Arabian Gulf are home to many fungal species that synthesize several enzymes, including laccases. In this study, we have screened fungi-producing laccases via the ABTS and BPB plate assays to facilitate the visual identification of potent laccase-positive isolates. Reaction halos around the growing colonies signified the potential of the isolated fungi in the oxidation of complex aromatic structures and stains [9] and emphasized that laccase-producing fungi are abundant in the sampling region.

By the completion of the screening study, the most potent laccase-producing isolates EK12, MLK46, EK56, EK59, EQ75, EK81, and EK107 were characterized till the species level. Initial morphological analysis was performed by microscopic examination and colony morphology inspection. In addition, the molecular analysis of *18SrRNA* gene, *5.8SrRNA* gene, and *28SrRNA* gene sequences using ITS1 and ITS2 was performed as it offers new in-depth insights into their genetic makeup and enhances our understanding of their evolutionary relationships. The *18SrDNA* gene has often been viewed as a marker of selection for identification. However, sequencing of the ITS rDNA piece has recently been considered a more precise tool for full characterization of fungi till the species level. The isolates were finally identified as *C. lunata* MLK46 (recovered from mangrove soil), *A. alternata* EK12 (from farm soil), *Acrophialophora levis* EK56 (from shoreline soil), *A. alternata* EK59 (from farm soil), *A. terreus* EQ75 (from yellow sponge siphon), *A. alternata* EK81 (from annual seablite soil), and *A. nidulans* EK107 (from shoreline soil). It is obvious that potent laccase-producing *A. alternata* is common in the sampling area, where three out of seven isolates were identified under this name. Laccase production from *A. alternata* and *A. terreus* was identified in some studies. In addition, *A. nidulans*-producing laccase was also identified. *C. lunata* MY3-producing laccase was also isolated from agricultural soil in Mansura, Egypt [2]. *Curvularia* sp.-producing laccase was similarly recovered from a soil sample in India [14]. However, according to our knowledge, laccase production from *Acrophialophora levis* was not identified before.

Due to its levels of laccase productivity and the ability to degrade BPB stain, *C. lunata* MLK46 was chosen for the next experiments. A series of cultural and nutritional factors were then evaluated to upstream its enzyme synthesis. The optimum fermentation pH was determined at pH 6.5, with an activity of 130.9 U/mL, and an optimal fermentation temperature was found at 30 °C, where laccase activity reached 141.7 U/mL. Furthermore, the productivity peaked with an incubation period of 5 d aligning with findings from similar research on marine-derived fungal isolates. According to previous studies, five marine fungi-producing laccases were isolated from the coastal areas of Tunisia, with the highest level in the case of *Trichoderma asperellum* (180–200 U/mL), while productivity from *Stemphylium lucomagnoense* and *A. nidulans* remained below 50 U/mL [1]. The best laccase level was found at 5–7 d in the case of *Botrytis cinerea* [15]. *C. lunata* MY3 produced the maximum level on the fifth day of incubation. However, some species [2] of fungi produced the best amounts of laccase after prolonged incubations (12–30 d) [2]. Fungal cultures from *Penicillium martensii* NRC 345 and *C. lunata* MY3 prefer acidic pHs for laccase production, which coincides with the current research [2]. *Curvularia* sp. maximally produced 60 U/mL of laccase at pH 4.0 and 30 °C [14].

To maximize the level of laccase production by *C. lunata* MLK46 recovered from mangrove forest soil at the Qatif Coast on the Arabian Gulf, we tested the effect of NaCl and the induction salts CuSO_4_ and MnSO_4_. These can affect both fungal development and the stability of secreted laccase by affecting the surface charges and conformational structure of protein molecules. In this research, a high level of secreted laccase was produced in the presence of a high level of NaCl as high as 1.5% in the culture medium (221.36 U/mL with 2.31-fold increase). Also, NaCl was found to enhance laccase production from the marine fungi *Cerrena unicolor* [16] and *Pestalotiopsis* sp. [17] isolated from mangroves. A similar effect was also observed with *T. asperellum* 1, with an optimal value at 75 h with 1.8 mM NaCl [1]. However, the enzyme of *Trematosphaeria mangrovei* lost half of its initial value in the occurrence of 1% NaCl [18]. Salt-tolerant laccases generally possess highly negative surface charges that might provide stability to the protein structure under severe osmolytic settings [19].

In most of the studies, CuSO_4_ was found to be a potent laccase inducer for various microorganisms [20]. In the current research, the best copper concentration was 0.2 mM, yielding about 257.83 U/mL laccase activity. This result coincides with *Polyporus brumalis* laccase production (0.25 mM, [21]). However, optimal copper concentration for laccase production from *T. asperellum* 1 [1] and *Pestalotiopsis* sp. [22] was 1.8 mM and 2.0 mM CuSO_4_, respectively. CuSO_4_ induction may be attributed to involvement of four copper atoms in the building of each laccase molecule. In addition, it was found that the occurrence of copper ions in the surrounding basal media induces laccase gene transcription. However, higher levels of copper may exert toxicity against the producing cells, because it adversely binds proteins, enzymes, nucleic acids, and other major functional molecules and metabolites [23].

Sodium nitrate served as the most suitable nitrogen source (286.5 U/mL), and galactose was determined to be the most effective carbon source (324.3 U/mL) for productivity from *C. lunata* MLK46. This can enhance the growth and development of the producer within the culture medium. Also, the fungus requires an additional, easily metabolizable carbon source to proceed with enzyme biosynthesis. In the study of laccase production from *T. asperellum* 1 [1] and *Arthrospira maxima* [4], sucrose was the best carbon source. These optimized conditions significantly increased laccase productivity, highlighting the importance of precise environmental and nutritional factors in maximizing laccase yield.

The SDS-PAGE analysis for the purified laccase from *C. lunata* MLK46 confirmed the successful purification procedure and provided an estimated molecular weight of 71.1 kDa. In respect to other investigators, our result is comparable with laccase from *Trichoderma harzianum* (72 kDa) [24]. MLK46 laccase has a lower value than laccases from *P. ostreatus* ARC280 (85 kDa) [25], *Trichoderma harzianum*, (79 kDa) [7], and *Marasmius* species BBKAV79 (75kDa) [26]. On the other hand, it has a higher value than laccases from *Agaricus sinodeliciosus* (69 kDa) [5], *Pleurotus ostreatus* HP-1 (68 kDa) [3], *Trametes velutina* JS18 (67 kDa) [27], *Abortiporus biennis* (66 kDa) [28], *C. lunata* MY3 (65 kDa) [2], *Aureobasidium pullulans* (61 kDa) [29], LCC1 of *P. ostreatus* V-184 (60 kDa) [30], LCC2 of *P. ostreatus* V-184 (65 kDa) [30], *Trametes versicolor* (50 kDa) [31], and *Trametes orientalis* (44 kDa) [32].

When MLK46 laccase was characterized biochemically, it exhibited a peak at pH 6.0, and the pH stability was highest between pH 6.0 and 9.0 during the exposure period (2 h). The high pH stability of the purified laccase from *C. lunata* MLK46 may be an interesting characteristic that could be used in specific biotechnological applications. Also, *Curvularia* sp. laccase was most active at pH 5.2–6.0 [14]. *C. lunata* MY3 was also active at acidic pH values, with pH 5.0 optimal for activity and pH 4.5–5.5 optimal for laccase stability [2]. *Ganoderma australe* laccase was stable within a pH range starting at 4.0 up to 7.0 [33]. The acidic behavior of MLK46 laccase coincides with the formerly produced enzymes from *C. lunata* MY3 [2], *Abortiporus biennis* J2 [28], *Thielavia* sp. [34], and *T. versicolor* [31], as they showed optimal activity at pH 5.0. The acidic behavior was also found in laccases from *C. kusanoi* L7 (pH 6.0) [35], *Trametes orientalis* (pH 4.0) [32], *Marasmius* sp. BBKAV79 (pH 5.5) [26], and *T. harzianum* WL1 (pH 4.5) [7]. The reason for the variable pH optima of laccases may be due to their dependence on the type of substrate used and the redox potential of the surrounding medium. The higher activity of laccases under acidic conditions may be because organic donors of protons (H^+^) are easily used as substrates. Additionally, the lower activity under high pH values may be due to the less ionization of substrates and the reduction of hydroxyl ions (OH^-^) binding with the active site of the enzyme [3,32,36].

MLK46 laccase showed maximal activity at 50 °C, which exactly coincides with other laccases from *Curvularia* sp. (50 °C) [14], *P. ostreatus* HP-1 (50 °C) [3], and *P. ostreatus* ARC280 (50 °C) [25]. However, it was higher than laccases produced from *C. lunata* MY3 (40 °C) [2], *C. kusanoi* (40 °C) [35], *Hericium coralloides* (40 °C) [37], *Marasmius* sp. BBKAV79 (40° C) [26], *T. Versicolor* (42.5 °C) [31], *T. harzianum* WL1 (35 °C) [7], and *P. ostreatus* (35 °C) [20]. On the other hand, the optimal temperature was lower than that reported for the laccase from *T. orientalis* (80 °C) [32].

In addition, MLK46 laccase showed remarkable thermal stability (50–80 °C) during the exposure period (1 h) and demonstrated exceptional longevity at 50 °C, with a *T*_1/2_ of 333.7 min. This behavior of thermal stability is better than those reported earlier. Laccases from *C. lunata* MY3 [2], *C. kusanoi* L7 [35], and *Streptomyces cyaneus* [38] almost conserved 75% of their initial activity after incubation at 50 °C for 120 min. It was also more stable than the purified laccase from *P. ostreatus* EM-1 where it retained 22.6% of its activity at 50 °C [39]. *Curvularia* sp. laccase was thermally stable below 50° C where the *T*_1/2_ at 50 °C was 24 min [14].

*C. lunata* MLK46 laccase was severely inhibited by 2-mercaptoethanol, L-cysteine, 1,4-DTT, and NaN_3_. This coincides with laccases from *C. lunata* MY3 [2], *Aureobasidium pullulans* NAC8 [40], *Trametes orientalis* [32], *Pleurotus* sp. [41], and LacA and LacB of *Trichoderma harzianum* S7113 [42]. In addition, EDTA and SDS showed moderate inhibition against MLK46 laccase, the same as LacA, LacB [42], and the laccase of *C. lunata* MY3 [2]. Interestingly, H_2_O_2_ enhanced our laccase activity, suggesting that *C. lunata* MLK46 laccase has unique regulatory properties, making it more adaptable under oxidative conditions. However, the ability of the tested enzyme to oxidize the tested substrates in the presence of H_2_O_2_ declares that it is not a peroxidase-like enzyme. The inhibitory activity of NaN_3_ against laccase may be attributed to its potential to direct the internal electron transport from their way to O_2_ reduction towards binding with the trinuclear copper center of the enzyme, leading to the inhibition of laccase-catalyzed oxidation reactions [43], while the inhibitory activity of EDTA may be attributed to its chelating potential to structural metallic ions located in the trinuclear copper center of laccase, which are responsible for its catalytic activity.

The tested enzyme exhibited high laccase activity in the presence of most metal ions, especially Fe^3+^ ions (1773.91 U/mL) and Ca^2+^ ions (1755.1 U/mL), which highlights its adaptability to most minerals. On the other hand, most of the laccase activity was lost at 8 mM of Hg^2+^ ions and 2 mM of Ag^+^ ions. This coincides with other laccases such as *C. lunata* MY3 laccase, which was enhanced by Mn^2+^ and Mg^2+^ ions, and inhibited by Hg^2+^ ions [2]. *Curvularia* sp. laccase was also inhibited by Ag^+^ and Hg^2+^ ions at concentrations as low as 0.1 mM. It was completely inhibited by Hg^2+^ ions at 20 mM. However, other ions also inhibited the activity with only 30–47% of measurable activity [14]. Also, Hg^2+^ ions drastically suppressed the activity of *C. lunata* MY3 enzyme at 10 mM [2]. Similar suppression was reported for laccases from *Marasmius* sp. BBKAV79 [26], *Xylaria* sp. [44], and *Sporothrix carnis* [45]. Stimulation of laccases by Cu^2+^ ions may be attributed to the presence of two kinds of active centers, the mononuclear copper center and the trinuclear copper center. The first is responsible for the oxidation of substrates, while the second copper centers are responsible for the reduction of molecular oxygen to water molecules. However, few laccases, such as those from *Thielavia* sp. [34] and *T. orientalis* [32], were not stimulated by Cu^2+^ ions.

Furthermore, the degradation potential of various substrates was evidenced by measuring the kinetic efficiency constant, which ranged from 40.95 to 238.20 mM^−1^ s^−1^. When ABTS, guaiacol, *o*-dianisidine, and 2,6-dichlorophenol were utilized as substrates, laccase exhibited the catalytic efficiency of 100.75, 151.20, 238.20, and 40.95 mM^−1^ s^−1^, respectively. This is significantly higher compared to laccase from *Meripilus giganteus,* which showed a *K*_cat_/*K*_m_ value against ABTS of 37 mM^−1^ s^−1^ [46], and better compared to the laccase from *Trametes polyzona* WRF03, where the catalytic efficiency of laccase against guaiacol, ABTS, and o-dianisidine was 2.11, 181.51, and 47.54 mM^−1^ s^−1^, respectively [6]. In addition, it was better than the laccase from *Pleurotus ostreatus* HP-1, which showed catalytic efficiency of 5.25, 0.52, 2.08, and 1.86 mM^−1^ s^−1^ against ABTS, 2,6-dimethoxyphenol, guaiacol, and *o*-dianisidine, respectively [24]. However, other laccases showed superior values, such as laccase Lcc2 of *Pleurotus pulmunarius,* which showed catalytic efficiency of 7238, 563, and 54,500 mM^−1^ s^−1^ against ABTS, guaiacol, and syringaldazine, respectively [47], and the laccase of *Pleurotus florida*, which showed the values of 4.13 × 107 and 3.31 × 105 mM^−1^ s^−1^ against *o*-dianisidine and guaiacol, respectively [48].

One of the most important aims of this research was to inspect the ability of MLK46 laccase to degrade various substrates by oxidative reactions. UV-visible spectrophotometry was used to analyze the substrate-specific reactions in the absence and presence of an enzyme. The tested laccase showed efficient oxidation peaks by electron transition against most of the tested compounds, especially guaiacol (at 300 nm), *o*-dianisidine (at 480 nm), ABTS (at 420 nm), and 2,6-dichlorophenol (at 600 nm), suggesting its strong potential as a biodegradation tool. In the existing literature, the laccase from *Cryphonectria parasitica* showed spectral transitions against ABTS and 2,4-dimethoxyphenol at 400–460 nm [49]. The laccase of *Trametes polyzona* WRF03 showed substrate-specific oxidation peaks against α-naphthol (at 510 nm) and guaiacol (at 460 nm) [6]. *C. lunata* MY3 [2] and *P. ostreatus* [50] laccases strongly oxidized ABTS while moderately oxidizing guaiacol. The preference of laccases to certain substrates differs according to the source strain, the type of side groups, and their position on the benzene ring. It is known that the oxidation of ABTS produces the ABTS azine cation radical (ABTS^·+^), which is then converted to the ABTS divalent cation (ABTS^2+^). For this, it turned to a blue color, which exhibited a characteristic peak at 420 nm [51]. Regarding other phenolic substrates, they lose one electron per molecule due to laccase oxidation, forming the aryloxy radical. Due to the non-stability of these radicals, they may be self-coupled or cross-coupled with other surrounding phenolic substrates, forming colored structures that give characteristic peaks at certain wavelengths [6,52].

## 5. Conclusions

This study aimed to screen the unexplored marine localities of Saudi Arabia for fungi-producing laccases with exceptional properties. Among various potent isolates, a promising laccase-producing fungus *C. lunata* MLK46 was chosen based on its potential to degrade both ABTS and BPB stain. It demonstrated exceptional properties over other reported fungal laccases, including production in the presence of 1.5% NaCl and a remarkable thermal stability at high temperatures (50–80 °C). Additionally, *C. lunata* MLK46 laccase exhibited enhanced activity in the presence of H_2_O_2_, highlighting its unique regulatory characteristics. Its kinetic analysis against ABTS, guaiacol, *o*-dianisidine, and 2,6-dichlorophenol demonstrated high catalytic efficiency ranging from 40.95 to 238.20 mM^−1^ s^−1^. Moreover, the spectral analysis demonstrated oxidation efficiency against these industrial substrates. These results collectively demonstrate the potential of MLK46 laccase as a promising agent in the treatment of polluted areas holding synthetic stains and biodegradation of various aromatic structures that demand strong enzymatic activity and compatibility. This provides a possible sustainable approach for managing industrial pollutants and advancing enzyme-based applications.

Future research should focus on scaling up its productivity in bioreactors to assess feasibility, operational stability, and cost-effectiveness. Advancements in enzyme engineering, such as immobilization techniques and directed evolution, could enhance stability, reusability, and substrate specificity, broadening its industrial applications. Additionally, molecular studies on *C. lunata* MLK46 laccase resilience under extreme conditions, such as salinity may provide key insights for engineering more robust biocatalysts tailored for diverse biotechnological and environmental applications.

## Figures and Tables

**Table 1:** The most potent laccase-producing fungal isolates. The assay was based on the spectrophotometric measurement of ABTS degradation at *A*_420_.

Isolate Code	Location	Source	Laccase Activity (U/mL)	Isolate Characterization	GenBank Accession Number
EK12	Jubail	Farm #2 with a history of pesticide application	66.52 ± 4.24	*Alternaria alternata*	PQ106852.1
MLK46	Qatif	Mangrove rhizosphere	295.65 ± 17.94	*Curvularia lunata*	PQ100161.1
EK56	Dammam	Shoreline #2	70.43 ± 6.42	*Acrophialophora levis*	PQ056709.1
EK59	Jabil	Farm #1 with a history of pesticide application	222.17 ± 12.42	*Alternaria alternata*	PQ056708.1
EQ75	Dhahran	Yellow sponge siphon	73.91 ± 6.23	*Aspergillus terreus*	PQ056704.1
EK81	Jubail	Annual seablite rhizosphere	193.04 ± 12.52	*Alternaria alternata*	PQ056707.1
EK107	Ras Tanura	Shoreline #1	140.86 ± 10.57	*Aspergillus nidulans*	PQ056706.1

**Table 2:** Brief of the optimized parameters for upstream processing of laccase productivity by *C. lunata* MLK46. The early production was performed in an enzyme production medium consisting of 0.10 g of yeast extract, 10.00 g of glucose, 0.22 g of (NH_4_)_2_SO_4_, 0.20 g of KH_2_PO_4_, 0.05 g of MgSO_4_, 0.50 g of KCl, 0.05 g of sodium molybdate, 0.03 g of FeSO_4_, 0.50 g of ZnSO_4_, and 0.08 g of CuSO_4_ per liter with pH adjusted at 5.50 and incubation at 30 °C for 5 d.

Parameter	Optimal Value	Maximal Productivity (U/mL)	Fold (x)
Unoptimized conditions	NA	95.65	1.00
Fermentation pH	6.5	130.87	1.37
Fermentation temperature	30 °C	141.74	1.48
Incubation period	120 h	211.73	2.21
NaCl	0.26 mM	221.36	2.31
MnSO_4_	0.3 mM	116.09	1.21
CuSO_4_	0.2 mM	257.83	2.70
FeCl_3_	61.65 mM	267.39	2.79
Nitrogen source	1.0% sodium nitrate	286.52	2.99
Carbon source	1.0% galactose	324.34	3.39

## Data Availability

The datasets in this research can be found in online repositories. The names of the repositories and accession numbers are included in this article.

## Abbreviations

The following abbreviations are used in this manuscript:

*T*_1/2_	Half-life time
1,4-DTT	1,4-dithiothreitol
ABTS	2,2′-azino-bis(3-ethylbenzothiazoline-6-sulfonic acid)
EDTA	Ethylenediamine-tetraacetate
*K*_cat_	Turnover number
*K*_cat_/*K*_m_	Kinetic efficiency constant
*K*_m_	Michaelis–Menten constant
NaN_3_	Sodium azide
PDA	Potato dextrose agar
SDS	Sodium dodecyl sulfate
SDS-PAGE	Sodium dodecyl sulfate polyacrylamide gel electrophoresis
*V*_max_	Maximum velocity of reaction
